# Romosozumab increases bone mineral density in postmenopausal Chinese women with osteoporosis: A randomised phase three study

**DOI:** 10.1016/j.jot.2026.101135

**Published:** 2026-06-03

**Authors:** Zhang Zhenlin, Xue Qingyun, Chen Decai, Chao Aijun, Huo Yanan, Zhu Mei, Cao Xu, Lin Hua, Xu Youjia, Cheng Qun, Yang Huilin, Xu Xiaoyan, Li Yujie, Du Xuan, Jiang Jun, Gao LingLing, Rosario-Jansen Theresa, Bauer Lars, Xia Weibo

**Affiliations:** a Shanghai Sixth People's Hospital, China; b Department of Orthopedics, Beijing Hospital, National Center of Gerontology, Beijing, China; c West China Hospital, Sichuan University, China; d Tianjin Hospital, China; e Jiangxi Provincial People's Hospital, China; f Department of Endocrinology and Metabolism, Tianjin Medical University General Hospital, Tianjin, China; g Sichuan Provincial People's Hospital, University of Electronic Science and Technology of China, Chengdu, China; h Nanjing Drum Tower Hospital, The Affiliated Hospital of Nanjing University Medical School, China; i The Second Affiliated Hospital of Soochow University, China; j Department of Osteoporosis and Bone Disease, Huadong Hospital Affiliated to Fudan University, China; k The First Affiliated Hospital of Soochow University, China; l UCB, Shanghai, China; m UCB, Morrisville, NC, USA; n UCB, Monheim/Rhein, Germany; o Department of Endocrinology, Key Laboratory of Endocrinology, National Commission of Health, State Key Laboratory for Complex, Severe and Rare Diseases, Peking Union Medical College Hospital, Chinese Academy of Medical Science, No. 1 Shuaifuyuan, Wangfujing Street, Dongcheng District, Beijing, 100730, China

**Keywords:** ‘Biomarkers’, ‘Bone remodelling’, ‘Clinical trial’, ‘Osteoporosis, Postmenopausal’, ‘Postmenopause’

## Abstract

**Background:**

Osteoporosis, most commonly affecting postmenopausal women, imposes a high medical and financial burden in China, highlighting the need for more effective treatments. This phase three, multicentre, randomised, double-blind, placebo-controlled study, conducted across 31 centres in mainland China, aimed to assess the efficacy, safety, and pharmacokinetic and pharmacodynamic profiles of romosozumab in Chinese women with postmenopausal osteoporosis at high risk of fracture (NCT05067335).

**Methods:**

Three hundred and twenty-seven patients were randomised 2:1 to romosozumab 210 mg (n = 218) or matched placebo (n = 109) for 6 months, followed by open-label romosozumab for 6 months in all patients. The Primary endpoint was the percentage change from baseline in bone mineral density (BMD) of the lumbar spine after 6 months of romosozumab treatment.

**Results:**

Baseline patient characteristics were well balanced. By month 6, the least-square mean (LSM [95% CI]) percentage change from baseline in BMD of the lumbar spine was significantly higher with romosozumab (9.81% [9.18%, 10.45%]) versus placebo (0.44% [–0.42%, 1.31%]), with a between-group difference of 9.37% (8.34%, 10.39%; p < 0.001). Significant improvements were also observed at the total hip (2.93% [2.53%, 3.33%] vs 0.07% [–0.49%, 0.63%]; difference: 2.86% [2.18%, 3.54%; p < 0.001]) and femoral neck (3.33% [2.65%, 4.01%] vs −0.15% [–0.94%, 0.65%]; difference: 3.48% [2.58%, 4.38%; p < 0.001]). In patients initially randomised to romosozumab, BMD continued to increase at all sites through month 12. With romosozumab, an initial large increase from baseline in the bone-forming marker procollagen type 1 N-telopeptide (P1NP) was observed, whilst the bone resorption marker serum type I collagen C-telopeptide (sCTX) decreased, demonstrating dual effects on bone formation and resorption. Romosozumab was well tolerated, with low incidence of serious and severe treatment-emergent adverse events (TEAEs), and few TEAEs leading to treatment or study discontinuation.

**Conclusion:**

These findings demonstrate the efficacy and safety of romosozumab for the treatment of osteoporosis in Chinese women with postmenopausal osteoporosis.

**The Translational Potential of this Article:**

In this phase three, multicentre, randomised, double-blind, placebo-controlled study, romosozumab treatment was effective and well tolerated in Chinese women with postmenopausal osteoporosis, being broadly consistent with findings observed outside of China. These results provide robust evidence to support the use of romosozumab in Chinese women with postmenopausal osteoporosis.

## Graphical abstract

**Figure:**
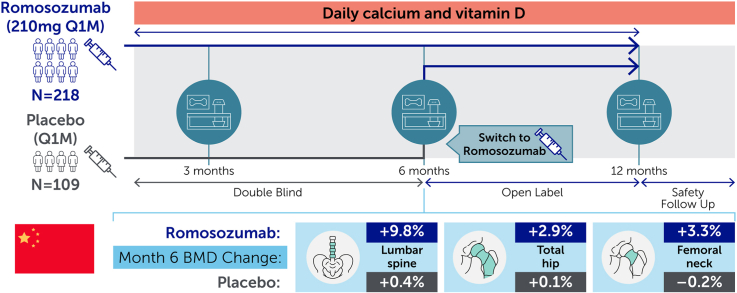

## 1 Introduction

Osteoporosis, most commonly affecting postmenopausal women, is characterised by low bone mass and bone microarchitecture deterioration, which together give rise to compromised bone strength and increased risk of fracture [1]. With an aging population in China, the number of individuals experiencing osteoporosis has risen significantly and presents an important public health issue [2]. According to a multicentre, cross-sectional study conducted in China from 2017 to 2018, the overall prevalence of osteoporosis in women aged 40 years or older was estimated to be 20.6%, with prevalence increasing with age [3]. Another study estimated a total population of approximately 70 million women aged over 50 years living with osteoporosis in China, representing 32.1% of this demographic [2]. Fractures due to osteoporosis impose a considerable burden. Epidemiological studies in China have reported vertebral fracture prevalence to be ∼15% in women over 50 years of age (4), 17.3% in women over 60 [4], and as high as 36.6% in women over 80 [5]. Research from another cross-sectional study in China indicated that vertebral fractures were prevalent in 24.2% of postmenopausal women with osteoporosis aged >50 years [6]. In a study which utilised hip fracture incidence as a surrogate index for osteoporotic fractures (osteoporosis combined with low-energy trauma being the predominant cause of hip fractures in people aged 55 years and above), an incidence of 177 hip fractures per 100,000 person-years in Chinese women aged over 55 was reported [7].

Critically, a previous fracture is a well-documented risk factor for future fractures [[8], [9], [10], [11]]. Indeed, the risk for subsequent fracture is highest immediately after an initial fracture [9,12,13], with reported incidences of 7.6% in the first year and 11.6% within the first two years post-fracture [14]. Collectively, these osteoporotic fractures, and osteoporosis more broadly, result in significant morbidity and mortality, and carry a high financial cost [2,[15], [16], [17]]. It is estimated that by 2035, expenditure on major osteoporotic fractures (i.e. wrist, vertebral body and hip) in China will reach 132 billion Chinese Yuan, equivalent to over 18 billion US dollars [2]. Consequently, there is an urgent need for effective osteoporosis management to rapidly reduce the risk of fractures in China.

Current pharmacological therapies recommended for osteoporosis in China by the *Guideline for diagnosis and treatment of primary osteoporosis (2022)* comprise five categories: bone resorption inhibitors, bone formation enhancers, dual-acting drugs, other mechanistic drugs, and traditional Chinese medicinal products [2]. Despite the availability of currently approved treatment options, there is a need in China for an approved treatment that can rapidly increase bone mass and strength to reduce fracture risk. This need is particularly acute in patients who have already suffered a fracture and are at high risk of future fractures [2].

Romosozumab is a humanised monoclonal antibody that binds to and inhibits sclerostin, exerting a dual effect on bone by increasing bone formation whilst decreasing bone resorption [[18], [19], [20]]. The multinational, phase 3 Fracture Study in Postmenopausal Women with Osteoporosis (FRAME; NCT01575834) demonstrated that one year of romosozumab treatment resulted in a significantly lower risk of vertebral and clinical fractures, with substantial gains in bone mineral density (BMD) at the lumbar spine, total hip, and femoral neck, compared with placebo [18]. Similarly, in a multinational, phase 3 study of postmenopausal women with osteoporosis who were at high risk for fracture (ARCH; NCT01631214), romosozumab treatment for 12 months followed by alendronate resulted in rapid gains in BMD at the same anatomical sites as in the FRAME trial, and significantly lower risk of fracture, including hip fracture, than alendronate alone [20]. In both ARCH and FRAME, the incidence of adverse events and serious adverse events were balanced between romosozumab and comparator treatment groups [18,20]. Based on the favourable results of these studies, romosozumab was approved in 2019 in Japan, the USA and Europe for the treatment of osteoporosis in postmenopausal women at high risk of fracture [21,22]. Despite the robust evidence supporting the efficacy and safety of romosozumab in postmenopausal women with osteoporosis [23], there is currently a lack of equivalent data in Chinese women, with most existing literature in East Asian populations being predominantly from Korean and Japanese cohorts. Data specific to the Chinese postmenopausal population are thus necessary to confirm consistent efficacy and safety and to inform prescribing practices in China.

The objective of this phase 3 study was to assess the efficacy, safety, pharmacodynamic and pharmacokinetic (PK) profiles of romosozumab treatment in Chinese women with postmenopausal osteoporosis, thus, bridging existing findings from outside of China.

## 2 Materials and methods

### 2.1 Study design

**Fig. 1:**
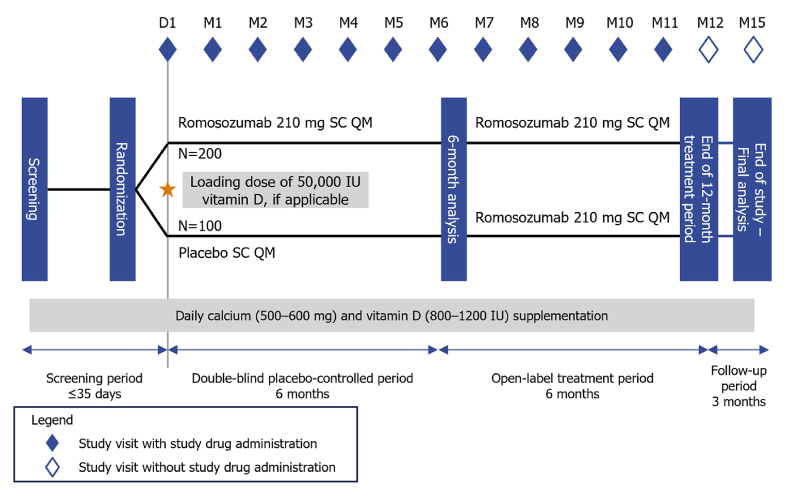
Study Design D: Day; IU: international units; M: Month; QM: every month; SC: subcutaneous.

This trial was a phase three, multicentre, randomised, double-blind, placebo-controlled study conducted across 31 centres in mainland China (NCT05067335; full trial protocol and statistical analysis plan available at clinicaltrials.gov). The study consisted of four periods: a screening period of 5 weeks, a 6-month double-blind placebo-controlled period, a 6-month open-label treatment period, and a 3-month safety follow-up period. The study design is presented in Fig. 1. During the double-blind, placebo-controlled period, patients were randomised 2:1 (stratified by age: <75 years; ≥75 years) to receive romosozumab 210 mg, or matched placebo, subcutaneously once every month (QM) for 6 months. Stratified block randomization, with a block size of six, was performed using an interactive response technology system, based on a predefined randomization list produced by a biostatistician not otherwise involved in the study. During the open-label treatment period, all patients received romosozumab 210 mg subcutaneously QM for 6 months but remained blinded to the original treatment received in the double-blind period. Throughout the study, patients were required to take daily oral calcium (500–600 mg) and vitamin D (800–1200 IU) supplementation.

### 2.2 Patients

Eligible patients were ambulatory, postmenopausal Chinese women, aged 55–90 years at the time of screening, with a BMD T-score of ≤ –2.50 at the lumbar spine, total hip, or femoral neck, as assessed by the central imaging vendor at the time of screening, based on dual-energy X-ray absorptiometry (DXA) scans, using reference data for Caucasian women from the National Health and Nutritional Examination Survey (NHANES, 1998) [24]. Patients also must have had at least one of the following independent risk factors for fracture: history of fragility fracture (except for: hip fracture, a severe [SQ3] vertebral fracture, or more than two moderate [SQ2] vertebral fractures), parental history of hip fracture, low body weight (body mass index ≤19 kg/m^2^), aged ≥65 years, or being a current smoker.

Patients must also have had at least two DXA-evaluable vertebrae in the lumbar 1–4 region and at least one DXA-evaluable hip. Patients were excluded if they had a BMD T-score of ≤ –3.50 at the total hip or femoral neck at screening, using reference data for Caucasian women from NHANES, 1998; had a history of myocardial infarction or stroke; had a known history of hip fracture; had any severe or more than two moderate vertebral fractures; or received prior osteoporosis treatments, including oral or intravenous bisphosphonates, denosumab, or teriparatide, within protocol-defined timeframes. Full inclusion and exclusion criteria are provided in Supplementary Material S1.

The study was conducted in accordance with the International Council for Harmonization (ICH)-Good Clinical Practice (GCP) requirements, the Declaration of Helsinki, applicable local laws and regulatory requirements, and was approved by ethics committees of participating sites (Supplementary Table 1). Informed consent was obtained from all individual participants included in the study.

### 2.3 Study procedures

Following screening, participants attended monthly study visits during the double-blind, placebo-controlled period (6 months) and the open-label treatment period (a further 6 months). The final visit took place at month 15 during the safety follow-up period. BMD measurements (DXA) at the lumbar spine, total hip, and femoral neck, were carried out at screening and months 3, 6, and 12. All DXA scans were analysed by the central imaging laboratory; analysts were blinded to patients’ treatment. Only Lunar or Hologic densitometers were allowed in the study. Adverse events were assessed over the entire study period. Blood samples for PK, antibodies to romosozumab, procollagen type 1 N-telopeptide (P1NP), and serum type I collagen C-telopeptide (sCTX) were drawn at Day 1, and months 1, 3, 6, 7, 9, and 12, with an additional sample for antibodies to romosozumab at month 15. Patients who discontinued study treatment were encouraged to complete the remaining study visits and assessments.

### 2.4 Outcomes

The primary endpoint was percentage change from baseline in BMD at the lumbar spine at the end of the double-blind, placebo-controlled period (month 6). The key secondary endpoints were percentage changes from baseline in BMD at the total hip and femoral neck at month 6 and percentage change from baseline in BMD at the lumbar spine, total hip, and femoral neck at the end of open-label treatment period (month 12) in patients initially randomised to romosozumab. Other efficacy endpoints were percentage changes from baseline in BMD at the total lumbar spine, hip and femoral neck at month 3. Subgroup analyses (stratifying by age group, geographical region, baseline BMD at each site, and baseline vitamin D level and loading dose of vitamin D) were also performed for the primary endpoint, to investigate the consistency and robustness of the treatment effect. Pharmacodynamic endpoints, including the percentage change from baseline in P1NP and sCTX at months 1, 3, 6, 7, 9, and 12, and pharmacokinetic endpoints of serum trough concentrations of romosozumab at baseline and at months 1, 3, 6, 7, 9, and 12 are also reported.

Treatment-emergent adverse events (TEAEs) through month 6 (the end of the double-blind, placebo-controlled period) and month 15 (the end of the safety follow-up period), are reported. TEAEs were coded using the Medical Dictionary for Regulatory Activities (MedDRA), version 24.1. TEAEs were graded as mild, moderate, or severe. TEAEs of interest included ‘injection site reactions’, ‘events potentially related to hypersensitivity’, ‘osteoarthritis’, ‘hyperostosis’, ‘malignancy’, ‘hypocalcaemia’, and ‘positively adjudicated osteonecrosis of the jaw, atypical femoral fracture, and cardiovascular events’. Serious adverse events were defined as those which caused death, were life-threatening, caused significant or persistent disability, or caused congenital anomaly/birth defects.

Additionally, the incidence of participants with anti-drug antibody (ADA) at baseline and through month 15 (end of safety follow-up), is reported.

### 2.5 Statistical analysis

The sample size was determined based on BMD data of all three anatomical sites from two phase three trials of romosozumab (NCT01575834 and NCT02016716 [unpublished]) [18,25]. A 2-group Satterthwaite t-test (2-sided, 5% significance level) was used. To ensure ≥90% power for all three BMD sites, the total required sample size was calculated to be 300 participants (200 in the romosozumab group and 100 in the placebo group).

All randomised patients were included in the randomised set (RS). Efficacy analyses used the full analysis set (FAS: comprising patients who received at least one dose of study treatment and provided at least one baseline and post-baseline BMD measurement). Safety analyses used the safety set (SS: comprising patients who received at least one dose of study treatment). Pharmacodynamic analyses were based on the pharmacodynamic per-protocol set (PD-PPS: all patients in the SS who had at least one evaluable bone turnover marker measurement with no further PD-related important protocol deviations). Pharmacokinetic analyses were based on the pharmacokinetic per-protocol set (PK-PPS: all patients in the SS who had at least one evaluable serum romosozumab concentration measurement with no further PK-related important protocol deviations).

The primary endpoint was evaluated using an analysis of covariance (ANCOVA) model, with treatment group, age strata group (stratification factor), baseline BMD value, machine type at baseline (Hologic or Lunar), region, and interaction of baseline BMD value and machine type at baseline as independent variables. Baseline values were defined as the latest measurement for a given patient up to and including the day of first study treatment administration. Statistical tests of efficacy endpoints are presented as 2-sided p-values; a fixed sequence testing procedure was used to handle multiple testing for the primary and secondary efficacy analyses, consisting of three steps [1]: lumbar spine BMD percentage change from baseline at 6 months [2], total hip BMD percentage change from baseline at 6 months, and [3] femoral neck BMD percentage change from baseline at 6 months. The statistical test of the treatment effects on step 2 was made in a confirmatory manner only when the treatment effect on step 1 was statistically significant using a 2-sided type I error rate of 0.05. Likewise step 3, was made only when the treatment effect tested in steps 1 and 2 was statistically significant. For efficacy analyses, observed post-baseline DXA BMD values after treatment discontinuation not due to coronavirus disease 2019 (COVID-19) or after alternative osteoporosis therapy were used as a treatment policy strategy. Missing or out of window post-baseline DXA BMD (>70 days since previous dose of investigational medicinal product) due to COVID-19 were imputed by multiple imputation using a missing at random approach. Missing post-baseline BMD values for reasons other than due to COVID-19 were imputed using the last observation carried forward approach.

Statistical analyses were performed using SAS version 9.4 or later.

## 3 Results

### 3.1 Patient disposition and baseline characteristics

**Fig. 2:**
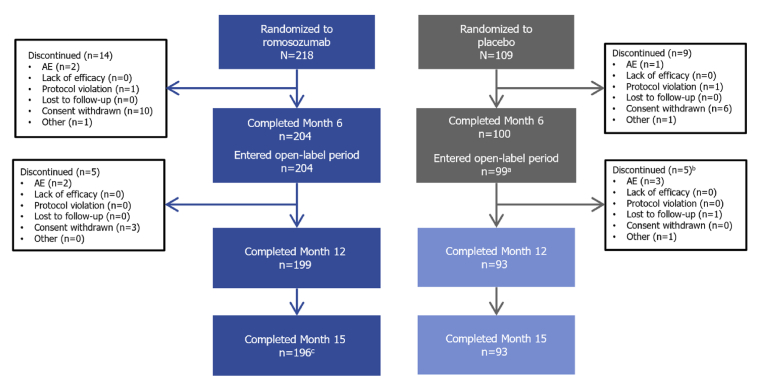
Patient disposition flow diagram ^a^One patient completed month 6 and discontinued due to an AE from month 6; ^b^One patient completed the double-blind, placebo-controlled period and planned to participate in the open-label period, but this patient did not receive open-label romosozumab at month 6 and then discontinued during the follow-up period. ^c^Three patients did not have the safety follow-up visit assessment; one patient died after completing their month 12 visit and two patients did not come back after their month 12 visit. AE: adverse event.

Between 21 October 2021 and 17 August 2022, 327 patients were randomised to romosozumab (n = 218) or placebo (n = 109) (Fig. 2). Of these, 93.6% (204/218) of patients in the romosozumab arm and 91.7% (100/109) of patients in the placebo arm completed the double-blind, placebo-controlled period; 91.3% (199/218) and 85.3% (93/109), respectively, completed the 12-month study period. For both the romosozumab (6.0% [13/218]) and placebo arms (5.5% [6/109]), the most common reason for study discontinuation during the 12-month study period was patient consent withdrawn.

Demographic and baseline characteristics were generally well-balanced (Table 1). The mean (standard deviation [SD]) age was 67.0 (5.7) years in the romosozumab arm and 67.3 (6.1) years in the placebo arm; body mass index was also comparable between arms (romosozumab: 23.6 [3.2] kg/m^2^ versus [vs] placebo: 23.0 [2.8] kg/m^2^). The mean (standard deviation; SD) BMD T-Score at the lumbar spine was equivalent across groups (romosozumab: –‍3.21 [0.74] vs placebo: −3.21 [0.65]). All patients were taking concomitant oral calcium and vitamin D supplementation at baseline, in accordance with the protocol. Approximately half of patients in both arms had prior protocol-permitted osteoporosis medication usage at baseline (romosozumab: 55.0% [120/218]; placebo: 50.5% [55/109]); the most common type being calcium carbonate/cholecalciferol (Table 1).

**Table 1:** Baseline demographics and characteristics.

Variable	Romosozumab (N = 218)	Placebo (N = 109)
**Mean age, years (SD)**	67.0 (5.7)	67.3 (6.1)
**Age, n (%)**		
55 to <65 years	63 (28.9)	31 (28.4)
65 to <85 years	154 (70.6)	76 (69.7)
≥85 years	1 (0.5)	2 (1.8)
**Sex, n (%)**		
Female	218 (100)	109 (100)
**Race, n (%)**		
Asian	218 (100)	109 (100)
**Weight, kg, mean (SD)**	56.5 (8.2)	55.7 (7.6)
**BMI, kg/m^2^, mean (SD)**	23.6 (3.2)	23.0 (2.8)
**Years since menopause, years, mean (SD)**	18.2 (7.0)	18.5 (7.0)
**BMD T-score at lumbar spine, mean (SD)**	−3.21 (0.74)	−3.21 (0.65)
**BMD T-score at total hip, mean (SD)**	−2.11 (0.59)	−2.13 (0.75)
**BMD T-score at femoral neck, mean (SD)**	−2.59 (0.55)	−2.55 (0.68)
**FRAX Score, %, mean (SD)**^a^	7.72 (2.87)	7.54 (3.06)
**Serum 25 OH vitamin D, ng/mL, mean (SD)**	29.82 (5.87)	30.09 (5.38)
**sCTX, μg/L, mean (SD)**	0.64 (0.36)	0.59 (0.34)
**P1NP, μg/L, mean (SD)**	61.86 (20.09)	58.34 (21.29)
**Prior Osteoporosis medication use, n (%)**	120 (55.0)	55 (50.5)
**Most common prior medication use, n (%)**^b^		
Calcium carbonate/cholecalciferol	52 (23.9)	25 (22.9)
Calcitriol	35 (16.1)	24 (22.0)
Cholecalciferol	24 (11.0)	7 (6.4)
Calcium carbonate	18 (8.3)	6 (5.5)
Vitamin D not otherwise specified	15 (6.9)	8 (7.3)
Alfacalcidol	11 (5.0)	2 (1.8)
**Previous fractures, n (%)**	77 (35.3)	42 (38.5)
**Most frequently reported fracture history site, n (%)**		
Spine	17 (7.8)	8 (7.3)
Wrist	10 (4.6)	10 (9.2)

Data are shown for the randomised set.

a 10-year probability of major osteoporotic fracture calculated with BMD; data are shown for the full analysis set (romosozumab: n = 214; placebo: n = 104).

b Only medications used in ≥5% of patients in either arm were included in this table. BMD: bone mineral density; BMI: body mass index; FRAX: Fracture Risk Assessment Tool; 25 OH vitamin D: 25-hydroxyvitamin D; P1NP: procollagen type 1 N-telopeptide; sCTX: serum type I collagen C-telopeptide; SD: standard deviation.

Due to the impact of COVID-19, 6.1% (20/327) of patients missed a visit and 46.2% (151/327) of patients experienced an out-of-window visit during the entire study period, with similar percentages in both arms (Supplementary Table 2). Only 0.9% (3/327) of patients missed the month 6 visit.

### 3.2 Primary endpoint: BMD changes

**Fig. 3:**
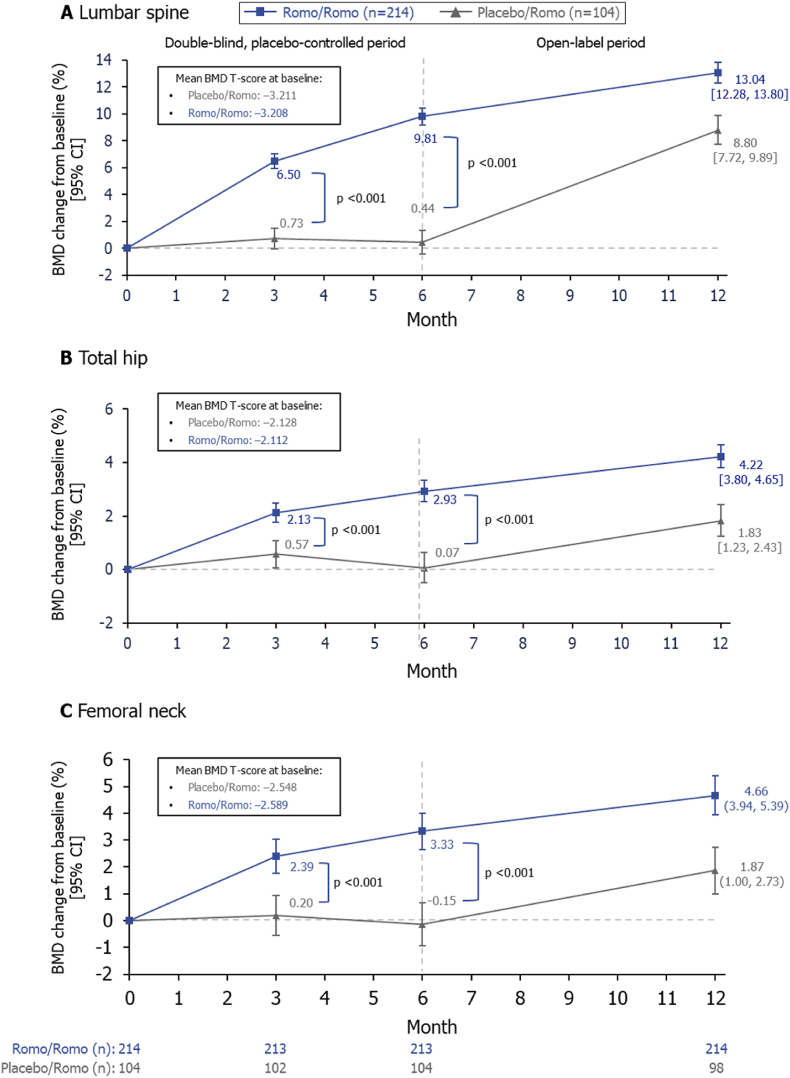
Least-square mean percentage changes from baseline in BMD at the (A) lumbar spine, (B) total hip, and (C) femoral neck Data are shown for the full analysis set. Month 3 p-values are exploratory and unadjusted and should thus be interpreted with caution. BMD: bone mineral density; CI: confidence interval; Romo: romosozumab.

At month 6, patients receiving romosozumab showed greater gains in BMD at all measured anatomical sites compared with placebo. In the romosozumab arm, there was a statistically significant improvement in BMD at the lumbar spine from baseline to month 6, with a least-square mean (LSM; 95% confidence interval [CI]) percentage change from baseline in BMD of 9.81% (9.18%, 10.45%), compared with placebo (0.44% [–0.42%, 1.31%]), resulting in a between-group difference of 9.37% (8.34%, 10.39%; p < 0.001) (Fig. 3a).

Similarly, by month 6, the LSM (95% CI) percentage change from baseline in total hip and femoral neck BMD was significantly greater in the romosozumab arm compared with the placebo arm. At the total hip, the changes were 2.93% (2.53%, 3.33%) vs 0.07% (−0.49%, 0.63%), respectively, with a between-group difference of 2.86% (2.18%, 3.54%; p < 0.001) (Fig. 3b). At the femoral neck, the changes were 3.33% (2.65%, 4.01%) vs −0.15% (−0.94%, 0.65%), respectively, with a between-group difference of 3.48% (2.58%, 4.38%; p < 0.001) (Fig. 3c).

Greater improvements in BMD at all three sites were apparent by month 3 with romosozumab treatment. The LSM (95% CI) percentage change from baseline in BMD was substantially higher in the romosozumab arm compared with the placebo arm (all between-group differences: p < 0.01 [nominal]) at the lumbar spine (6.50% [5.95%, 7.05%] vs 0.73% [–0.04%, 1.50%]; Fig. 3a), total hip (2.13% [1.77%, 2.48%] vs 0.57% [0.07%, 1.07%]; Fig. 3b), and femoral neck (2.39% [1.76%, 3.02%] vs 0.20% [–0.54%, 0.94%]; Fig. 3c).

Between month 6 and month 12, patients initially randomised to romosozumab demonstrated further increases in BMD. Patients initially randomised to placebo experienced generally similar increases in BMD from month 6 to month 12 with romosozumab treatment to those seen in the first 6 months in romosozumab-randomised patients (Fig. 3). By month 12, LSM (95% CI) percentage changes from baseline in BMD in patients initially randomised to romosozumab and those initially randomised to placebo were 13.04% (12.28%, 13.80%) and 8.80% (7.72%, 9.89%), respectively, at the lumbar spine (Fig. 3a), and 4.22% (3.80%, 4.65%) and 1.83% (1.23%, 2.43%) at the total hip (Fig. 3b), with similar findings at the femoral neck (Fig. 3c).

Subgroup analyses (stratifying by age group, geographical region, baseline BMD at each site, and baseline vitamin D level and loading dose of vitamin D) demonstrated generally consistent efficacy across subgroups, except for those with baseline vitamin D levels >40 ng/mL and those aged ≥75. As the small sample sizes for these two subgroups meant that changes could not be reliably measured, these results should be interpreted with caution (Supplementary Table 3).

### 3.3 Pharmacodynamic results

Levels of the bone turnover markers P1NP and sCTX were similar between the two arms at baseline (Table 1). In patients initially randomised to romosozumab, the highest median (interquartile range [IQR]) percentage change from baseline in P1NP was at month 1 (54.60% [52.20%]). After month 1, P1NP levels in these patients began to decrease through month 12. In patients initially randomised to placebo, the highest median (IQR) percentage change from baseline in P1NP was at month 7 (54.05% [58.80%]), which was one month after starting romosozumab. After month 7, P1NP levels in these patients began to decline and continued to decrease through month 12 (Supplementary Fig. 1a). In patients initially randomised to romosozumab, the lowest median [IQR] percentage change from baseline in sCTX occurred at month 12 (−42.40% [42.10]). In patients initially randomised to placebo, the lowest median [IQR] percentage change from baseline in sCTX was seen at month 7 (−31.60% [51.50]; Supplementary Fig. 1b).

### 3.4 Pharmacokinetic results

Romosozumab serum trough concentrations for study participants initially randomised to romosozumab increased through month 6, after which serum trough concentrations remained similar through month 12 (Supplementary Table 4).

### 3.5 Immunogenicity

At any time, 38.5% of participants initially randomised to romosozumab were ADA positive during the 15-month study, and 3.7% had neutralising antibodies. In total, 35.3% of participants initially randomised to romosozumab became treatment-emergent ADA positive up to month 15. Post-baseline, the number of patients initially randomised to romosozumab who experienced a first occurrence of ADA positivity generally increased at each visit through month 6 (Table 3). Among romosozumab-treated patients who developed ADA positivity, the first occurrence was most commonly observed at month 6 (16.5% [34/206]), with 30.6% (63/206) of patients in the romosozumab arm being positive for ADA at that time point (Table 3). Proportions of patients with ADA positivity at each subsequent visit was relatively similar through end of the study (month 15). The first occurrence of neutralising antibody positivity was observed at month 12 in patients initially randomised to romosozumab.

**Table 2:** Summary of TEAEs during the double-blind, placebo-controlled period and the entire study period (through month 15).

Item, n (%) [number of events]	Double-blind, placebo-controlled period (months 0–6)		Overall period (months 0–15)
	Romosozumab/romosozumab (N = 218)	Placebo/romosozumab^a^ (N = 109)	Romosozumab total^a^ (N = 316)
**Any TEAEs**	159 (72.9) [469]	77 (70.6) [200]	271 (85.8) [1324]
**Serious TEAEs**^b^	8 (3.7) [10]	5 (4.6) [5]	34 (10.8) [40]
**Patient discontinuations due to TEAEs**	2 (0.9) [2]	3 (2.8) [3]	7 (2.2) [7]
**TEAEs leading to treatment discontinuation**	2 (0.9) [2]	3 (2.8) [3]	8 (2.5) [8]
**Treatment-related TEAEs**	57 (26.1) [111]	21 (19.3) [34]	85 (26.9) [179]
**Severe TEAEs**	5 (2.3) [5]	1 (0.9) [1]	18 (5.7) [25]
**TEAEs leading to death**	0	0	1 (0.3) [1]
**Most common TEAEs**^c^			
COVID-19	15 (6.9) [15]	12 (11.0) [12]	71 (22.5) [72]
Suspected COVID-19	11 (5.0) [11]	4 (3.7) [4]	57 (18.0) [57]
Upper respiratory tract infection	13 (6.0) [16]	5 (4.6) [5]	37 (11.7) [46]
Cough	NR	NR	31 (9.8) [32]
Arthralgia	11 (5.0) [13]	5 (4.6) [5]	23 (7.3) [26]
Injection site reaction	19 (8.7) [27]	1 (0.9) [1]	26 (8.2) [39]
Pyrexia	NR	NR	22 (7.0) [22]
Back pain	8 (3.7) [9]	3 (2.8) [3]	17 (5.4) [19]
Hyperlipidaemia	9 (4.1) [9]	2 (1.8) [2]	16 (5.1) [16]
SARS-CoV-2 test positive	NR	NR	15 (4.7) [15]
Nasopharyngitis	8 (3.7) [8]	1 (0.9) [1]	13 (4.1) [15]
Constipation	7 (3.2) [7]	5 (4.6) [5]	12 (3.8) [12]
Hypertension	NR	NR	12 (3.8) [14]
Pain in extremity	7 (3.2) [9]	1 (0.9) [2]	11 (3.5) [14]
Blood glucose increased	NR	NR	11 (3.5) [15]
Leukopenia	NR	NR	11 (3.5) [16]
Pneumonia	NR	NR	10 (3.2) [10]
Hepatic steatosis	NR	NR	10 (3.2) [10]
Bronchitis	NR	NR	10 (3.2) [12]
Pulmonary mass	NR	NR	10 (3.2) [10]
Dizziness	6 (2.8) [6]	5 (4.6) [5]	9 (2.8) [13]
Diarrhoea	NR	NR	9 (2.8) [9]
Intervertebral disc protrusion	NR	NR	9 (2.8) [13]
Abdominal discomfort	NR	NR	9 (2.8) [9]
Pharyngitis	NR	NR	9 (2.8) [11]
Urinary tract infection	NR	NR	9 (2.8) [9]
Dermatitis	NR	NR	8 (2.5) [9]
Hypokalaemia	NR	NR	8 (2.5) [8]
Injection site pain	7 (3.2) [10]	1 (0.9) [1]	7 (2.2) [13]
Chronic gastritis	NR	NR	7 (2.2) [7]
Gastroesophageal reflux disease	NR	NR	7 (2.2) [9]
Rhinitis	NR	NR	6 (1.9) [6]
Lacunar infarction	NR	NR	5 (1.6) [5]
Gastritis erosive	NR	NR	3 (0.9) [3]

Data are shown for the safety set. TEAEs were coded using the MedDRA version 24.1.

a Only TEAEs within the romosozumab exposure period are included (i.e. following at least one dose of romosozumab).

b A serious TEAE was defined as a TEAE that met one or more of the following criteria: death; life-threatening; significant or persistent disability/incapacity; congenital anomaly/birth defect; important medical event that, based upon appropriate medical judgment, may have jeopardised the patient or study participant, and may have required medical or surgical intervention to prevent one of the other outcomes listed in the definition of serious; or initial inpatient hospitalization or prolongation of hospitalization.

c TEAEs reported by ≥ 3 patients in either treatment arm, by Preferred Term. NR: not reported; TEAE: treatment-emergent adverse event.

**Table 3:** Anti-romosozumab and neutralising antibody status and first occurrence of positive ADA results.

Visit	Anti-romosozumab and neutralising antibody status		First occurrence of positive ADA results
	Interpretation	Romosozumab/romosozumab N = 218 n/Nsub (%)	Romosozumab/romosozumab N = 218 n/Nsub (%)
**Baseline**	Positive	8/218 (3.7)	8/218 (3.7)
	Neutralising	0/218	
	Negative	210/218 (96.3)	
**Visit 3 (month 1)**	Positive	3/213 (1.4)	1/213 (0.5)
	Neutralising	0/213	
	Negative	210/213 (98.6)	
**Visit 5 (month 3)**	Positive	27/206 (13.1)	26/206 (12.6)
	Neutralising	0/206	
	Negative	179/206 (86.9)	
**Visit 8 (month 6)**	Positive	63/206 (30.6)	34/206 (16.5)
	Neutralising	0/206	
	Negative	143/206 (69.4)	
**Visit 14 (month 12)**	Positive	55/200 (27.5)	3/200 (1.5)
	Neutralising	2/200 (1.0)	
	Negative	145/200 (72.5)	
**Safetyfollow-up**	Positive	45/194 (23.2)	2/194 (1.0)
	Neutralising	7/194 (3.6)	
	Negative	149/194 (76.8)	
**Any time**	Positive	85/218 (38.5)	
	Neutralising	8/218 (3.7)	
	Negative	124/218 (61.5)	

Data are shown for the safety set. n refers to the number of study participants with relevant positive anti-romosozumab antibody status at the given visit. Nsub refers to the number of study participants with an assessment at the given visit. ADA: anti-drug antibody.

Participants that tested ADA positive at any time tended to have ∼30% lower mean serum trough romosozumab concentrations at month 3 relative to those that tested ADA negative throughout the study (Supplementary Fig. 2). The impact of ADA status on trough concentrations at months 9 and 12, however, was very limited, with 95% CIs for ADA positive and ADA negative study participants overlapping fully.

Patients with treatment-emergent ADA positivity showed very similar mean percent changes from baseline in BMD at the lumbar spine as patients in all other ADA subcategories (including ADA negative participants; Supplementary Fig. 3).

### 3.6 Safety

Safety data are reported for the double-blind, placebo-controlled period (through month 6) and for the entire study period (through month 15; Table 2). During the entire study period, the mean (SD) cumulative duration of exposure to romosozumab was 11.5 (2.4) months for patients initially randomised to romosozumab and 5.7 (1.1) months for those initially randomised to placebo. Overall, patients initially randomised to romosozumab had 208 patient exposure years, while those initially randomised to placebo had 46 patient exposure years. The mean number of doses of romosozumab received by patients in the two groups were 11.2 (2.3) and 5.6 (1.0), respectively. Overall treatment compliance, defined as the ratio of the number of injections received to the number of injections expected, was (mean [SD]) 98.9% [3.8%] for patients initially randomised to romosozumab and 95.9% [12.0%] for those initially randomised to placebo, with 99.1% and 91.8% of patients in each respective group having ≥80% treatment compliance.

During the double-blind, placebo-controlled period, TEAEs were experienced by 72.9% (159/218) of patients randomised to romosozumab, and by 70.6% (77/109) of patients randomised to placebo (Table 2), with an incidence rate of 263.45 (CI: 224.09, 307.73) per 100 patient-years at risk. In patients randomised to romosozumab, the proportion of patients who had ≥1 serious TEAE by month 6 was 3.7% (8/218) vs 4.6% (5/109) for placebo-randomised patients; serious TEAEs that were considered treatment related were reported in 0.5% (1/218) and 1.8% (2/109) of patients, respectively. The incidence of TEAEs leading to treatment discontinuation was 0.9% (2/218) vs 2.8% (3/109) for romosozumab and placebo randomised patients, respectively. TEAEs graded as severe were experienced by 2.3% (5/218) of patients treated with romosozumab and 0.9% (1/109) of patients treated with placebo. No deaths were reported in either arm during the double-blind period.

The most common TEAEs during the double-blind, placebo-controlled period (reported in ≥3% of patients in either arm; Table 2) included COVID-19, which was reported in 6.9% (15/218) and 11.0% (12/109) of patients treated with romosozumab and placebo, respectively, injection site reaction (reported in 8.7% [19/218] and 0.9% [1/109] of patients, respectively), and upper respiratory tract infection (reported in 6.0% [13/218] and 4.6% [5/109] of patients, respectively). Suspected COVID-19 was also reported for 5.0% (11/218) and 3.7% (4/109) of patients, respectively.

TEAEs of interest during the double-blind, placebo-controlled period included those potentially related to hypersensitivity, which were reported in 6.0% (13/218) and 9.2% (10/109) of patients treated with romosozumab and placebo, respectively. Hypocalcaemia was reported for one romosozumab-treated patient during the double-blind, placebo-controlled period. This TEAE was lab-confirmed, mild, did not lead to discontinuation of study treatment, and resolved.

In the romosozumab-arm, 0.9% (2/218) of patients experienced positively adjudicated cardiovascular TEAEs during the double-blind, placebo-controlled period (Supplementary Table 5), including one acute myocardial infarction TEAE and one coronary artery disease TEAE; no such events were reported in the placebo arm. Owing to low event numbers, statistical comparison between the two treatment groups was not feasible. No TEAEs for positively adjudicated osteonecrosis of the jaw or positively adjudicated atypical femoral fracture were reported in either arm during the double-blind, placebo-controlled period.

The collective incidence of treatment-emergent injection site reaction and hypersensitivity adverse events during the double-blind, placebo-controlled period was similar between placebo-randomised participants (70.6%) and romosozumab-randomised participants who were either ADA negative (72.1%), pre-existing ADA positive (75.0%) or developed ADA positivity (77.4%) and those with any form of ADA positivity (76.8%). The specific incidence of treatment-emergent injection site reaction (by MedDRA preferred term) was higher among romosozumab-randomised participants with any form of ADA positivity (13.0%), those who developed ADA positivity (14.5%) and those with pre-existing ADA positivity (12.5%) compared with ADA negative participants (6.8%) and placebo-randomised participants (0.9%).

Over the entire study period, TEAEs were experienced by 85.8% (271/316) of patients who received ≥1 dose of romosozumab (Table 2), with an incidence rate of 265.69 (CI: 235.00, 299.28) per 100 patient-years at risk. Overall, the incidence of serious TEAEs was 10.8% (34/316) in patients who received ≥1 dose of romosozumab; 1.6% (5/316) of patients experienced a serious TEAE that was considered treatment-related. In patients who received ≥1 dose of romosozumab, the incidence of TEAEs leading to treatment discontinuation was 2.5% (8/316). Overall, 5.7% (18/316) of patients who received ≥1 dose of romosozumab experienced TEAEs graded as severe. During the entire study period, one death (cerebral infarction) was reported in patients who received ≥1 dose of romosozumab (1/316 [0.3%]). The event occurred 5 days after receiving the last dose of romosozumab 210 mg and was deemed not related to romosozumab treatment by the Investigator.

The most common TEAEs during the entire study period are reported in Table 2. Over the entire study period, TEAEs of fractures in patients who received ≥1 dose of romosozumab were low, with rib (0.9% [3/316]) and ankle (0.6% [2/316]) fractures being the most common.

Over the entire study period, comprising both patients initially randomised to romosozumab and to placebo, a total of 1.6% (5/316) of patients experienced positively adjudicated cardiovascular events, which are detailed in Supplementary Table 6.

## 4 Discussion

In this study, the first double-blind, placebo-controlled, phase three trial of romosozumab in Chinese women with postmenopausal osteoporosis, after 6 months of treatment with romosozumab, patients demonstrated significant improvements from baseline in BMD at the lumbar spine, total hip, and femoral neck compared with placebo. Notably, smaller improvements at all three sites were observed by the first post-baseline assessment at month 3, demonstrating a rapid response to treatment. Subgroup analyses reinforced these findings, with broadly similar efficacy at month 6 across baseline BMD levels, though small sample size in some subgroups limited further comparison. From month 6 to month 12, patients initially randomised to romosozumab treatment continued to experience further improvements in BMD at all measured sites.

The BMD changes observed at the lumbar spine and femoral neck in the present study are in line with those seen in the ARCH and FRAME global phase three studies of romosozumab, including a sub-analysis of East Asian patients in the ARCH trial [26], demonstrating a comparable level of efficacy in postmenopausal women with osteoporosis in China as reported outside of China [18,20]. Furthermore, the magnitude of BMD gains at months 6 and 12 were consistent with those reported in other studies of romosozumab's efficacy in East Asian populations [[27], [28], [29]]. Thus, this study robustly expands previous efficacy findings in East Asian populations from predominantly Korean and Japanese cohorts to Chinese postmenopausal women.

Importantly, in ARCH and FRAME, these BMD gains were associated with significant and rapid reductions in fracture risk [18,20]. BMD gains of this magnitude have been shown to be effective for the reduction of fracture risk, as demonstrated by the recent Foundation for the National Institutes of Health (FNIH)-American Society for Bone and Mineral Research (ASBMR)-Study to Advance BMD as a Regulatory Endpoint (FNIH-ASBMR-SABRE) project, which found a strong relationship between hip BMD and fracture risk [30]. Pertinently, based on the FNIH-ASBMR-SABRE project's findings, the Food and Drug Administration (FDA) have since (December 2025) qualified treatment-related change in total hip BMD as a surrogate endpoint for fracture in clinical trials of therapies for post-menopausal women with osteoporosis at risk for fracture [31]. While the overall rate of anti-romosozumab antibody positivity in the present study was higher than in ARCH and FRAME (38.5% vs 15.3% and 18.0%, respectively), akin to those studies, we observed no apparent association between ADA status and BMD change at the lumbar spine.

In the ARCH and FRAME studies, serum concentration of the bone formation maker P1NP [32] increased rapidly in the first month of romosozumab treatment and returned towards baseline levels by approximately month 6 [18,20]; the results described herein are consistent with this overall pattern and with other studies of romosozumab in Korean and Japanese cohorts [27,29]. Similarly, both the pattern and magnitude of change in serum concentration of the bone resorption marker sCTX [32] in the present study were also similar to the changes seen in the ARCH and FRAME and East Asian studies [18,20,27,29]. In this study, patients initially randomised to romosozumab experienced a sharp initial decrease in concentration, followed by a return to near-baseline levels by month 3, and a subsequent gradual decline through month 12. Thus, the impact of romosozumab treatment on bone turnover markers reported here is broadly aligned with those reported previously, supporting romosozumab's dual effect of increasing bone formation and decreasing bone resorption in this population.

It should be noted that while patients in the present study had baseline 25-hydroxyvitamin D levels that were moderately higher than reported mean values for postmenopausal women in mainland China [33], and generally similar to those observed in a similar European study [34], the present study excluded those with vitamin D insufficiency (<20 ng/mL). Thus, comparisons to population-level studies are limited and it is not possible to compare the impact of the dietary pattern of patients in this study to those from outside of China. Furthermore, while baseline P1NP and sCTX concentrations were in line with reference ranges for postmenopausal Chinese women [35], few studies have compared racial differences in bone remodelling and its contribution to skeletal health, precluding meaningful comparisons to studies outside of mainland China [36].

Treatment with romosozumab was well tolerated, with safety findings that were generally consistent with the known safety profile of romosozumab, and no new safety concerns identified [18,20]. In both arms, the incidence of TEAEs graded severe, as well as the incidence of serious TEAEs, was low and few TEAEs led to treatment or study discontinuation; the incidences were generally similar to those observed in the ARCH and FRAME trials [18,20]. In the present study, 0.9% of patients randomised to romosozumab experienced adjudicated-positive cardiovascular serious adverse events during the double-blind placebo-controlled period (0% in placebo-randomised patients). In the global ARCH and FRAME trials, any adjudicated-positive cardiovascular serious adverse events occurred in 2.5% and 1.3% of romosozumab-randomised patients during their respective controlled periods (exposure-adjusted incidence rates [EAIR] per 100 participant years at risk: 2.6 and 1.4, respectively). EAIR for cardiovascular categories in the present study were not calculated and thus cannot be directly compared. The interpretation of adjudicated cardiovascular event data is limited by the small overall sample size; the 2:1 randomization ratio employed, and thus the larger sample size in the romosozumab arm during the first 6 months, inherently increased the likelihood of observing such events. More broadly, due to the shorter duration of the controlled period in in the present study (6 months) compared with other studies (up to 12 months) and the smaller sample size employed compared with global study populations, comparisons of safety data between the present study and existing studies from outside of China should be interpreted with caution.

This study's key strength was its double-blind, placebo-controlled design, which minimised potential confounders and allowed for an unbiased comparison between romosozumab and placebo. However, several limitations must be acknowledged. Firstly, while the study provided evidence on the effect of romosozumab on BMD and bone turnover marker changes, the study was not designed to evaluate whether romosozumab confers benefits in reducing osteoporotic fractures in Chinese women with postmenopausal osteoporosis. Nevertheless, the ARCH and FRAME studies have already robustly evidenced the fracture-risk-reduction efficacy of romosozumab in postmenopausal women with osteoporosis, which is complemented by the FNIH-ASBMR SABRE Project's validation of a BMD surrogate threshold effect for fracture risk reduction, culminating in the FDA's recent qualification of treatment-related change in total hip BMD as a surrogate endpoint for fracture [31,37]. While substantial BMD gains were observed in this study, fracture-risk reduction in the Chinese population has not yet been directly demonstrated.

Secondly, this study was conducted during the COVID-19 pandemic in China, which impacted patient attendance and the timing of assessments. However, while almost half of patients had out-of-window visits, only 6.1% of patients had missing visits, and just 6.7% had a visit >70 days outside of the window. Therefore, despite disruption of the visit schedule, the overall impact of COVID-19 on efficacy assessment in this study was likely limited.

The findings of this study may have implications for clinical practice in China. Across East Asia, osteoporosis may be underdiagnosed and undertreated, with an increasing burden of imminent fracture risk, especially in China [26,38,39]. Chinese patients may therefore be diagnosed with osteoporosis later in the disease course, strengthening the need for therapies that can quickly restore microarchitecture and BMD. Thus, the rapid BMD gains observed in this study, alongside a safety profile that was generally consistent with global studies, highlights the potential benefit of romosozumab for high-risk patients.

In conclusion, in this study of Chinese women with postmenopausal osteoporosis, treatment with romosozumab demonstrated significant improvements from baseline in BMD (lumbar spine, total hip, and femoral neck) after 6 months compared with placebo. While fracture risk reduction was not assessed in the present study, these positive changes in BMD were similar to those reported in previous, large, global, phase three studies from outside of China that also established the fracture-risk-reduction efficacy of romosozumab which formed the basis for its approval in the USA, Europe, and Japan. Romosozumab treatment was well tolerated in the postmenopausal Chinese patient population, with safety findings consistent with the established safety profile observed both in global studies and in studies of East Asian cohorts. These results provide robust evidence to support the efficacy and safety of romosozumab for the treatment of osteoporosis in Chinese women with postmenopausal osteoporosis.

## Informed consent

Informed consent was obtained from all individual participants included in the study.

## Ethical approval

All procedures performed in studies involving human participants were in accordance with the ethical standards of the institutional and/or national research committee and with the 1964 Helsinki declaration and its later amendments or comparable ethical standards. Ethical approval was provided by the ethics committees of participating sites (Supplementary Table 1). Informed consent was obtained from all individual participants included in the study.

## Authors’ contributions

Substantial contributions to study conception/design, or acquisition/analysis/interpretation of data: **ZZ, QX, DC, AC, YH, MZ, XC, HL, YX, QC, HY, XX, YL, XD, JJ, LLG, TRJ, LB, WX**; Drafting of the publication, or reviewing it critically for important intellectual content: **ZZ, QX, DC, AC, YH, MZ, XC, HL, YX, QC, HY, XX, YL, XD, JJ, LLG, TRJ, LB, WX**; Final approval of the publication: **ZZ, QX, DC, AC, YH, MZ, XC, HL, YX, QC, HY, XX, YL, XD, JJ, LLG, TRJ, LB, WX**.

## Clinical trial registration

NCT05067335.

## Declaration of generative AI in scientific writing

No generative artificial intelligence (AI) or AI-assisted technologies were used in the preparation of this manuscript.

## Funding

This study was sponsored by UCB and 10.13039/100002429Amgen Inc. Support for third-party writing assistance for this article, provided by Ning Li, B.Eng., Costello Medical, 10.13039/100007472UK, and Andrew Wilhelmsen, PhD, Costello Medical, 10.13039/100007472UK, was funded by 10.13039/100011110UCB in accordance with Good Publication Practice (GPP 2022) guidelines (https://www.ismpp.org/gpp-2022).

## Conflicts of interest

**ZZ:** None disclosed; **QX:** None; **DC:** None disclosed; **AC:** None; **YH:** None; **MZ:** None; **XC:** None; **HL:** None disclosed; **YX:** None disclosed; **QC:** None; **HY:** None; **XX:** Employee and stockholder of UCB; **YL:** Employee and stockholder of UCB; **XD:** Employee of UCB; **JJ:** Employee of UCB; **LLG:** Employee of UCB; **TRJ:** Employee and stockholder of UCB; **LB:** Employee and stockholder of UCB; **WX:** None.

## Data Availability

Underlying data from this manuscript may be requested by qualified researchers six months after product approval in China, and 18 months after trial completion. Investigators may request access to anonymised individual patient-level data and redacted trial documents which may include: analysis-ready datasets, study protocol, annotated case report form, statistical analysis plan, dataset specifications, and clinical study report. Prior to use of the data, proposals need to be approved by an independent review panel at www.Vivli.org and a signed data sharing agreement will need to be executed. All documents are available in English only, for a pre-specified time, typically 12 months, on a password protected portal.
