# Supplementary material for: Romosozumab increases bone mineral density in postmenopausal Chinese women with osteoporosis: A randomised phase three study
Source: J Orthop Translat. 2026 Jun 3;59:101135. doi: 10.1016/j.jot.2026.101135 (PMC13251483; doi:10.1016/j.jot.2026.101135)
Supplement: Multimedia component 1 [file mmc1.pdf]

Supplementary Material

Romosozumab Increases Bone Mineral Density in Postmenopausal Chinese Women with Osteoporosis: A Randomized Phase 3 Study

Zhenlin Zhang, MD,[a] Qingyun Xue, MD, PhD,[b] Decai Chen, MD,[c] Aijun Chao, MSc,[d] Yanan Huo, MB,[e] Mei Zhu, MD, PhD,[f] Xu Cao, MD,[g] Hua Lin,MD,[h] Youjia Xu, MD, PhD,[i] Qun Cheng, MD, PhD,[10] Huilin Yang, MD, PhD,[11] Xiaoyan Xu, MSc,[12] Yujie Li, MD,[12] Xuan Du, MD, PhD,[12] Jun Jiang, PhD,[12] LingLing Gao, BSc,[12] Theresa Rosario-Jansen, PhD,[13] Lars Bauer, MD,[14] Weibo Xia, MD[15]

Zhang Zhenlin (zzl2002@medmail.com.cn),[a] Xue Qingyun (xueqingyun2018@163.com),[b] Chen Decai (18980601309@163.com),[c] Chao Aijun (ajoinmail@sohu.com),[d] Huo Yanan (13970029871@126.com),[e] Zhu Mei (meichuqin@163.com),[f] Cao Xu (caoxu97036@126.com),[g] Lin Hua (lh2116@126.com),[h] Xu Youjia (xuyoujia@suda.edu.cn),[i] Cheng Qun (quncheng_2014@163.com),[j] Yang Huilin (suzhouspine@163.com),[k] Xu Xiaoyan (Annie.Xu@ucb.com),[l] Li Yujie (Yujie.Li@ucb.com),[l] Du Xuan (Xuan.Du@ucb.com),[l] Jiang Jun (derekjuang1973@hotmail.com),[l] Gao LingLing (Lingling.Gao@ucb.com),[l] Rosario-Jansen Theresa (Theresa.Rosario-Jansen@ucb.com),[m] Bauer Lars (Lars.Bauer@ucb.com),[n] **Xia Weibo** (xiaweibo8301@163.com)[o]

[a]Shanghai Sixth People's Hospital, China

[b]Department of Orthopedics, Beijing Hospital, National Center of Gerontology, Beijing, China

[c]West China Hospital, Sichuan University, China
[d]Tianjin Hospital, China
[e]Jiangxi Provincial People's Hospital, China
[f]Department of Endocrinology and Metabolism, Tianjin Medical University General Hospital, Tianjin, China
[g]Sichuan Provincial People's Hospital, University of Electronic Science and Technology of China, Chengdu, China
[h]Nanjing Drum Tower Hospital, the Affiliated hospital of Nanjing University Medical School, China
[i]The Second Affiliated Hospital of Soochow University, China
[j]Department of Osteoporosis and Bone Disease, Huadong Hospital Affiliated to Fudan University, China
[k]The First Affiliated Hospital of Soochow University, China
[l]UCB, Shanghai, China
[m]UCB, Morrisville, NC, USA
[n]UCB, Monheim/Rhein, Germany
[o]Department of Endocrinology, Key Laboratory of Endocrinology, National Commission of Health, State Key Laboratory for Complex, Severe and Rare Diseases, Peking Union Medical College

Hospital, Chinese Academy of Medical Science, No. 1 Shuaifuyuan, Wangfujing Street, Dongcheng District, Beijing 100730, China

Correspondence to: Weibo Xia, No. 1 Shuaifuyuan, Wangfujing Street, Dongcheng District, Beijing 100730, China. Email: xiaweibo8301@163.com; Tel: +86 69151552.

Clinical trial Registration: NCT05067335

Supplementary Material S1

Inclusion Criteria:

To be eligible to participate in this study, all of the following criteria must have been met:

1. An independent review board/independent ethics committee approved written informed consent form was signed and dated by the study participants or legal representative prior to initiation of any study specific activities/procedures.

2. Study participant was considered reliable and capable of adhering to the protocol, visit schedule, and medication intake according to the judgment of the investigator.

3a. Study participant was an ambulatory postmenopausal Chinese woman, 55 to 90 years of age (inclusive) at the time of screening. Postmenopausal was defined as no spontaneous vaginal bleeding or spotting for 12 or more consecutive months prior to screening.

4. Study participant must have had a BMD T-score ≤–2.50 at the lumbar spine, total hip, or femoral neck, as assessed by the central imaging vendor at the time of screening based on DXA scans, and using data for Caucasian women from the National Health and Nutritional Examination Survey (NHANES, 1998).

5. Study participant must have had at least 1 of the following independent risk factors for fracture:

- History of fragility fracture (except hip fracture, a severe [SQ3] vertebral fracture or more than 2 moderate [SQ2] vertebral fractures [see exclusion criteria])
- Parental history of hip fracture
- Low body weight (body mass index ≤19 kg/m^2)
- Elderly (age ≥65 years)
- Current smoker

6. Study participant must have had at least 2 vertebrae in the lumbar 1 to lumbar 4 region and at least 1 hip that were evaluable by DXA, as assessed by the central imaging vendor.

Exclusion Criteria:

Study participants were not permitted to enroll in the study if any of the following criteria were met:

1. Study participant had a BMD T-score of ≤–3.50 at the total hip or femoral neck, as assessed by the central imaging vendor at the time of screening based on DXA scans and using data for Caucasian women from NHANES 1998.

2. Study participant had a known history of hip fracture.

3. Study participant had any severe (SQ3) or more than 2 moderate (SQ2) vertebral fractures, as assessed by the central imaging vendor based on the lateral spine x-ray at screening (Visit 1).

4. Study participant had a history of myocardial infarction.

5. Study participant had a history of stroke.

6. Study participant had a vitamin D insufficiency, defined as 25 (OH) vitamin D levels <20ng/mL, as assessed by the central laboratory at screening. Vitamin D repletion was to be permitted and the study participant may have been retested once within the screening period.

7. Study participant used oral bisphosphonates:

- Any doses received within 3 months prior to randomization (Day 1)
- More than 1 month of cumulative use between 3 and 12 months prior to randomization

− More than 3 years of cumulative use, unless the last dose was received ≥5 years prior to randomization

8. Study participant used intravenous (iv) bisphosphonates:

− Zoledronic acid

◦ Any doses received within 3 years prior to randomization

◦ More than 1 dose received within 5 years prior to randomization

− Intravenous ibandronate, iv pamidronate, or iv alendronate (ALN)

◦ Any doses received within 12 months prior to randomization

◦ More than 3 years of cumulative use, unless the last dose was received ≥5 years prior to randomization

9. Study participant used denosumab or any cathepsin K inhibitor:

− Any doses received within 18 months prior to randomization

10. Study participant used tibolone, cinacalcet, or calcitonin:

− Any doses received within 3 months prior to randomization

11. Study participant used teriparatide (TPTD) or any parathyroid hormone (PTH) derivative:

− Any doses received within 3 months prior to randomization

− More than 1 month of cumulative use between 3 and 12 months prior to randomization

12. Study participant used systemic oral or transdermal estrogen or selective estrogen receptor modulators (SERMs):

− More than 1 month of cumulative use within 6 months prior to randomization

13. Study participant used strontium ranelate or fluoride:

− More than 1 month of cumulative use within 5 years prior to randomization

14. Study participant used hormonal ablation therapy:

− More than 1 month of cumulative use within 6 months prior to randomization

15. Study participant used systemic glucocorticosteroids:

− ≥5mg prednisone equivalent per day for more than 14 days within 3 months prior to randomization

16. Study participant had a history of metabolic or bone disease (except osteoporosis) that may have interfered with the interpretation of the results, such as sclerosteosis, Paget's disease, rheumatoid arthritis, osteomalacia, osteogenesis imperfecta, osteopetrosis, ankylosing spondylitis, Cushing's disease, hyperprolactinemia, and malabsorption syndrome.

17. Study participant had a history of solid organ or bone marrow transplants.

18. Study participant had a history of ONJ or AFF.

19. Study participant had a confirmed diagnosis or was under investigation for multiple myeloma or related lymphoproliferative disorder at the screening Visit.

20. Study participant had evidence of any of the following:

a. Current, uncontrolled hyper- or hypothyroidism. Uncontrolled hyperthyroidism was defined as thyroid-stimulating hormone (TSH) and thyroxine (T4) outside of the normal range. Uncontrolled hypothyroidism was defined as TSH >10.

b. Current, uncontrolled hyperparathyroidism or history of hypoparathyroidism. Uncontrolled hyperparathyroidism was defined as PTH outside the normal range in study participants with concurrent hypercalcemia or PTH values >20% above upper limit of normal (ULN) in normocalcemic study participants.

c. Current hypercalcemia or hypocalcemia, defined as albumin-adjusted serum calcium outside the normal range, as assessed by the central laboratory at the time of screening. Albumin-adjusted serum calcium levels could be retested once in the case of an elevated albumin-adjusted serum calcium level within 1.1xULN of the laboratory's reference ranges.

d. Study participant had ≥3xULN of any of the following: alanine aminotransferase (ALT), aspartate aminotransferase (AST), alkaline phosphatase (ALP), or >ULN total bilirubin (≥1.5xULN total bilirubin if known Gilbert's syndrome). If the study participant had elevations only in total bilirubin that were >ULN and <1.5xULN, fractionated bilirubin was used to identify possible undiagnosed Gilbert's syndrome (i.e. direct bilirubin <35%).

For randomized study participants with a baseline result >ULN for ALT, AST, ALP, or total bilirubin, a baseline diagnosis and/or the cause of any clinically meaningful elevation was understood and recorded in the eCRF.

If a study participant had >ULN ALT, AST, or ALP that did not meet the exclusion limit at screening, the tests were repeated, if possible, prior to dosing to ensure there was no further ongoing clinically relevant increase. In case of a clinically relevant increase, inclusion of the study participant was discussed with the Medical Monitor.

21. Study participant was currently receiving treatment in another investigational device or drug study, or less than 30 days or 5 half-lives (whichever is longer) since ending treatment on another investigational device or drug study(ies).

22. Study participant was undergoing other investigational procedures while participating in this study.

23. Study participant had previously entered this study or had previously participated in a study with a sclerostin antibody product.

24. Study participant had a malignancy within the last 5 years, except nonmelanoma skin cancers or cervical or breast ductal carcinoma in situ.

25. Study participant had a known hypersensitivity to any of the products to be administered during dosing (calcium supplements, vitamin D products, or mammalian cell-derived products).

26. Study participants who were breastfeeding or planned to become pregnant or breastfeed during the study or within 12 weeks of the final dose of IMP.

27. Study participant had a history or evidence of any other clinically significant disorder, condition, or disease (e.g. untreated or unstable, with the exception of those outlined above) that, in the opinion of the investigator, if consulted, posed a risk to study participant safety or interfered with the study evaluation, procedures, or completion.

28. Study participant showed a positive result for human immunodeficiency virus (HIV), hepatitis C virus, or hepatitis B infection at screening (Visit 1).

29. Study participant had an active tuberculosis infection within 6 months of signing the ICF.

30. Study participant had active pneumonia.

31. Study participant had a reported history of hearing loss associated with cranial nerve VIII compression due to excessive bone growth (e.g. as seen in conditions such as Paget's disease, sclerosteosis, and osteopetrosis).

Supplementary Fig 1 Median ± interquartile range of percentage changes from baseline in (A) P1NP (bone formation marker), and (B) sCTX (bone resorption marker)

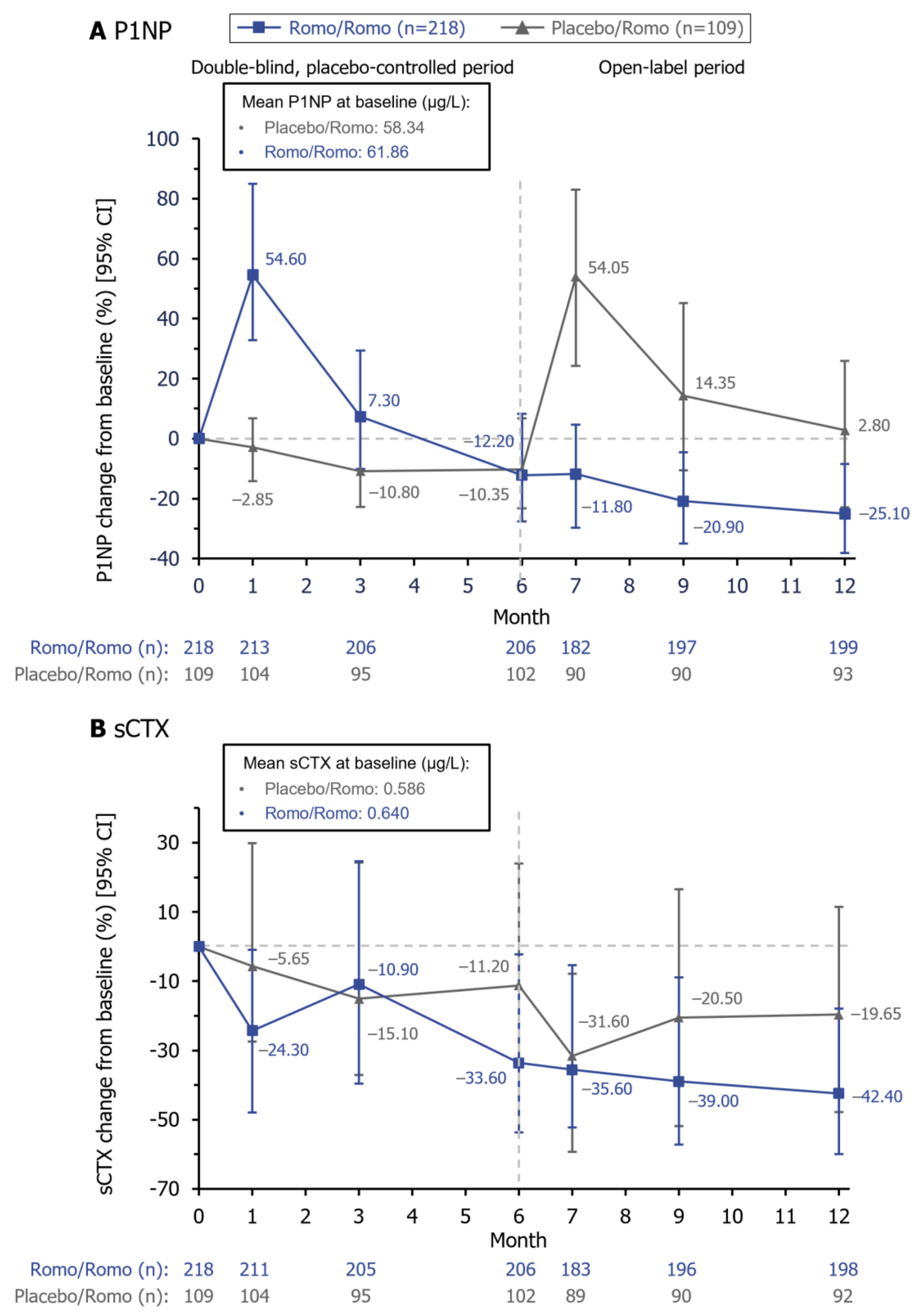

Data are shown for the pharmacodynamic per-protocol set. P1NP: procollagen type 1 N-telopeptide; sCTX: serum type I collagen C-telopeptide.

Supplementary Fig 2 Geometric Mean (95% CI) serum trough romosozumab concentration in the romosozumab/romosozumab group by cumulative anti-romosozumab antibody status

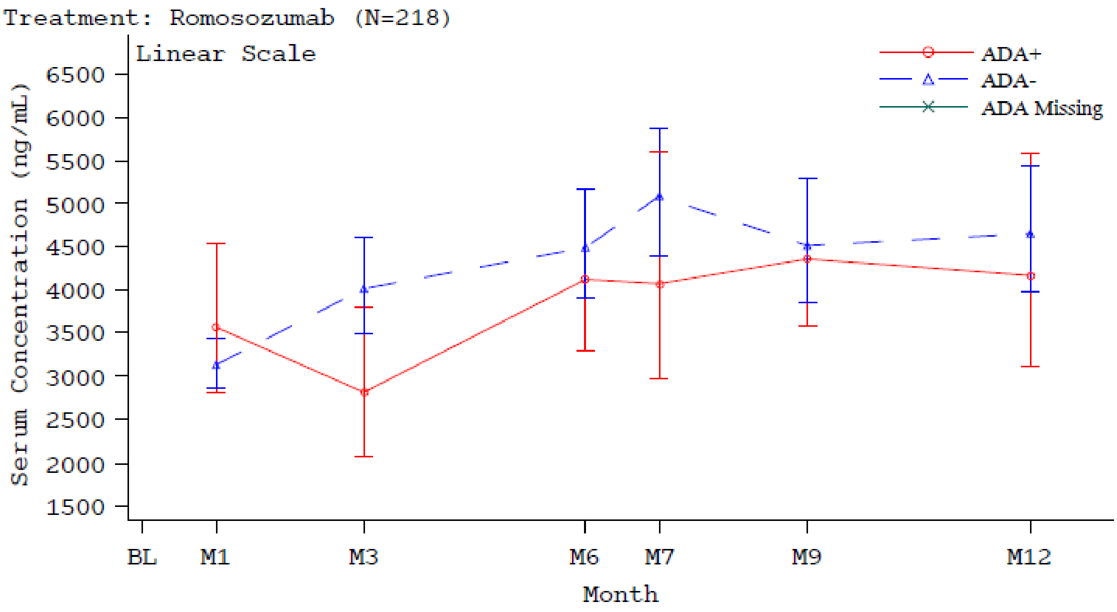

Data are shown for the pharmacokinetic per-protocol set. Values BLQ were replaced by the value of LLOQ/2=25ng/mL in the calculations of geometric mean and confidence intervals. The LLOQ was (50ng/mL). Data are only displayed if at least two-thirds of the data were above LLOQ at the respective timepoint. The ADA status (positive or negative or missing/inconclusive) was considered in a cumulative manner at each timepoint; a study participant was counted positive from the first visit at which the study participant achieved a positive ADA sample result to the end of the treatment period (regardless of any missing/inconclusive or negative ADA sample result after the positive record); if a study participant had only negative ADA samples or only one missing/inconclusive sample with all other samples as ADA negative up to that timepoint, the study participant was classified as negative; otherwise, the study participant was classified in the missing ADA category. There were no study participants in the "ADA Missing" category. ADA: anti-drug antibody; BL: baseline; BLQ: below limit of quantitation; CI=confidence interval; LLOQ: lower limit of quantification; M: month.

Supplementary Fig 3 Percent change from Baseline in BMD at the lumbar spine by visit and ADA subcategory to month 12

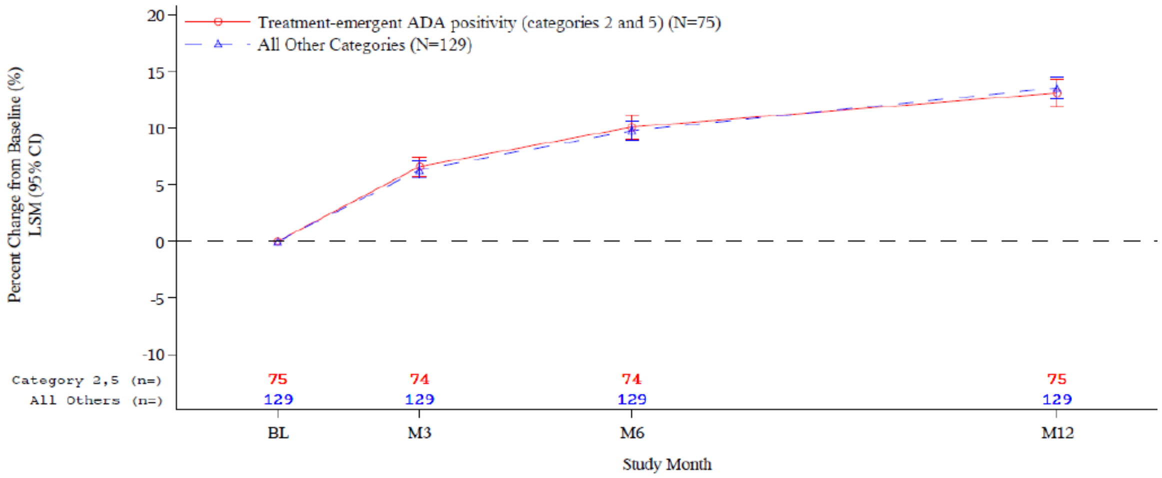

Data are shown for the full analysis set. ADA: anti-drug antibody; BL: baseline; BMD: bone mineral density; M: month.

Supplementary Table 1. List of study sites, ethics committees/institutional review boards and dates of approvals for OP0002 Protocol Amendment 2.

Study Site	Ethics Committee/Institutional Review Board	Reference Number	Date of Approval
Peking Union Medical College Hospital	Drug Clinical Trial Ethics Committee<br>Peking Union Medical College Hospital,<br>Chinese Academy of Medical Sciences<br>No. 1 Shuaifuyuan, Dongcheng District, Beijing<br>CHINA	KS2020157	2020/4/21
Beijing Hospital	Ethics Committee of Beijing Hospital<br>No. 1 Dongdan Dahua Road, Dongcheng District, Beijing 100730<br>CHINA	2020BJYYEC-138-02	2020/11/4
Shanghai Sixth People's Hospital	Ethics Committee of Shanghai Sixth People's Hospital<br>No. 600, Yishan Road, Shanghai<br>CHINA	2020-028	2020/5/5
Guangdong Provincial People's Hospital	Medical Ethics Committee of Guangdong Provincial People's Hospital,<br>23rd Floor, Haiyin Center, 98 East South Road, Yuexiu District, Guangzhou, Guangdong<br>CHINA	YW2020-022-04	2020/7/24
Huadong Hospital Affiliated to Fudan University	Ethics Committee of Huadong Hospital Affiliated to Fudan University<br>No.221, Yan'an west road, Jing 'an district, Shanghai<br>CHINA	20200079	2021/1/27
The Second Affiliated Hospital of Soochow University	Ethics Committee of The Second Affiliated Hospital of Soochow University<br>No.1055, Sanxiang Road, Soochow, Jiangsu<br>CHINA	JD-LS-2020-008-03	2020/5/25

Affiliated Union Hospital of Tongji Medical College, Huazhong University of Science and Technology (Wuhan Union Hospital)	Clinical Trial Ethics Committee of Huazhong University of Science and Technology<br>No.13 Hanghang Road, Wuhan City, Hubei Province<br>CHINA	[2020] Ethics Approval No. (13)-1	2020/5/27
Shanghai General Hospital	Ethics Committee of Shanghai General Hospital<br>No. 85/86, Wujin Road, Hongkou District, Shanghai<br>CHINA	Y Ethics Review [2021] No. 015	2021/2/3
Tianjin Hospital	Ethics Committee of Tianjin Hospital<br>No. 406 Jiefang South Road, Hexi District, Tianjin<br>CHINA	(2020) Ethical Review No. YW-002	2020/6/15
West China Hospital of Sichuan University	Ethics Committee on Clinical Trial West China Hospital of Sichuan University<br>No.37 Guoxue Lane, Wuhou District, Chengdu City, Sichuan Province<br>CHINA	NA	2020/9/2
Shanghai Ninth People's Hospital, Shanghai JiaoTong University School of Medicine	Medical Ethics Committee of Shanghai Ninth People's Hospital<br>No. 639 Zhizaoju Road, Shanghai<br>CHINA	SH9H-2020-C48-2	2021/1/29
The First Affiliated Hospital of Jinan University (Guangzhou Overseas Chinese Hospital)	Clinical Trial Ethics Committee of the First Affiliated Hospital of Jinan University<br>No.613, Huangpu Avenue West, Tianhe District, Guangzhou, Guangdong<br>CHINA	[2019] Ethical Review Approval Drug No.021-1	2020/5/27
Beijing Tsinghua Changgung Hospital	Ethics Committee of Beijing Tsinghua Changgung Hospital<br>No.168, Litang Road, Changping District, Beijing<br>CHINA	19234-1-02<br>19234-1-03	2020/6/16
Shanghai Tenth People's Hospital (Tenth People's Hospital of Tongji University)	Ethics Committee of Shanghai Tenth People's Hospital<br>No. 301 Middle Yanchang Road, Shanghai<br>CHINA	SHSY-IEC-4.1/20-37/03	2020/7/17
Beijing Luhe Hospital, Capital Medical University	Medical Ethics Committee of Beijing Luhe Hospital, Capital Medical University<br>No.82, Xinhua South Road, Tongzhou District, Beijing<br>CHINA	2020-LHYW-006-01	2020/6/3

Peking University First Hospital	Peking University First Hospital Biomedical Research Ethics Committee<br>Peking University First Hospital, No.8, Xishiku Street, Xicheng District, Beijing, 100034<br>CHINA	2020 Drug Registration 025	2020/7/29
Tongji Hospital of Tongji University	Ethics Committee of Shanghai Tongji Hospital<br>No.389 Xincun Road, Putuo District, Shanghai<br>CHINA	(Tongji) EC Review No.2020-020	2020/5/27
Beijing Pinggu Hospital	Medical Ethics Committee of Beijing Pinggu Hospital<br>No.59, Xinping North Road, Pinggu District, Beijing, 101299<br>CHINA	2020-Y 005-01	2020/8/31
Jishuitan Hospital	Ethics Committee of Beijing Ji Shui Tan Hospital<br>No.31 Xinjiekou East Street, Xicheng District, Beijing<br>CHINA	J.T.E.R. No. 20200501	2020/5/13
The First hospital affiliated to Zhengzhou University	The Ethics Committee of Scientify Research and Clinical Trial,<br>The First Affiliated Hospital of Zhengzhou University<br>Jianshe East Road 1, Zhengzhou, Henan<br>CHINA	Drug-2020-046	2020/6/23
Sichuan Provincial People's Hospital	Sichuan Academy of Medical Sciences - Sichuan Provincial People's Hospital Ethics Committee<br>for Clinical Trials of Medicines and Medical Devices<br>No. 32, West Second Section, 1st Ring Road, Chengdu City, Sichuan Province<br>CHINA	Ethical Review (Drug) 2020 No.13-1	2020/7/20
The First People's Hospital of Yueyang	Clinical Trial Ethics Committee of Yueyang Central Hospital<br>No.39 Dongmaoling Road, Yueyang, Hunan, China/ No.28 Yueyang Road, Yueyang, Hunan<br>CHINA	Yueyang No.1 People’s Hospital Ethics Review Drug Clinical No. (2020-006)	2020/5/6
West China Hospital of Sichuan University	Ethics Committee on Clinical Trial West China Hospital of Sichuan University<br>No.37 Guoxue Lane, Wuhou District, Chengdu City, Sichuan Province<br>CHINA	2020 Clinical Trial (Western	2020/9/24

		Medicine) Review (No.112)	
Nanjing Drum Tower Hospital, the Affiliated hospital of Nanjing University Medical School	Medical ethics committee of the Affiliated Hospital of Nanjing University Medical School<br>No. 321, Zhongshan Road,Nanjing, Jiangsu<br>CHINA	2020-045-02	2020/5/28
Tianjin Medical University General Hospital	Medical ethics committee of Tianjin Medical University General Hospital<br>154 Anshan Road, Heping District, Tianjin<br>CHINA	IRB2020-023-02	2020/4/17
Peking University Third Hospital	Peking University Third Hospital Medical Science Research Ethics Committee<br>No. 49 North Huayuan Road, Haidian District, Beijing<br>CHINA	(2020) Yao Lun Shen No.(057-01)	2020/7/31
Zhejiang Rui'an People's Hospital	Ethics Committee of Ruian People's Hospital<br>No.108 Wansong Road, Ruian, Zhejiang<br>CHINA	YW2020011	2021/8/2
Pingxiang People's Hospital	Ethics Committee of Pingxiang People’s Hospital<br>No.8, Wugongshan Middle Road, Pingxiang, Jiangxi<br>CHINA	2020Y041-KS02	2021/1/25
The 1st Affiliated hospital of Soochow University	Ethics Committee of The First Affiliated Hospital of Soochow University<br>No.899, Pinghai Road, Soochow, Jiangsu<br>CHINA	(2021) Ethical Approval No.052	2021/3/29
Shunde Hospital of Southern Medical University	Medical Ethics Committee of Shunde Hospital<br>Southern Medical University, No.1, Jiazi Road, Licun, Lunjiao Street, Shunde District, Foshan, Guangdong<br>CHINA	Ke Yan Lun Shen 20210211	2021/2/26
Jiangxi Provincial People's Hospital	Ethics Committee of Jiangxi Provincial People’s Hospital<br>No. 152, Aiguo Road, Nanchang, Jiangxi Province, 330006<br>CHINA	Yao Hui No. (2021) 11	2021/3/10

Dates correspond to the approval of Protocol Amendment 2 (14 Jan 2020).

Supplementary Table 2. Impact of COVID-19 on patient visits

Category	Romosozumab /romosozumab (N= 218)	Placebo /romosozumab (N=109)
Visit not conducted, total n (%)	12 (5.5)	8 (7.3)
Month 1	3 (1.4)	0
Month 2	4 (1.8)	3 (2.8)
Month 3	3 (1.4)	5 (4.6)
Month 4	2 (0.9)	0
Month 5	2 (0.9)	0
Month 6	2 (0.9)	1 (0.9)
Month 7	1 (0.5)	0
Month 9	1 (0.5)	0
Month 10	0	1 (0.9)
Month 11	0	1 (0.9)
Visit out of window[a], total n (%)	100 (45.9)	51 (46.8)
Day 1	5 (2.3)	5 (4.6)
Month 1	2 (0.9)	2 (1.8)
Month 2	5 (2.3)	1 (0.9)
Month 3	14 (6.4)	6 (5.5)
Month 4	21 (9.6)	14 (12.8)
Month 5	32 (14.7)	18 (16.5)
Month 6	32 (14.7)	24 (22.0)
Month 7	37 (17.0)	21 (19.3)
Month 8	45 (20.6)	19 (17.4)
Month 9	40 (18.3)	20 (18.3)
Month 10	38 (17.4)	20 (18.3)
Month 11	39 (17.9)	18 (16.5)
Month 12	34 (15.6)	16 (14.7)
Safety follow-up	7 (3.2)	4 (3.7)
>70 days	15 (6.9)	7 (6.4)

Data are shown for the randomized set. [a]A visit out of window is defined as a visit that deviates by more than 7 days from the originally scheduled date, either prior to or subsequent to the planned time.

Supplementary Table 3. Subgroup analyses: percent change from Baseline in BMD at the lumbar spine assessed by DXA at the end of the double-blind, placebo-controlled period (month 6)

Subgroup	Placebo, n; Romosozumab, n	Difference in LSM (romosozumab – placebo) Percent change from Baseline, (SE)[a]	95% CI
Age: <75 years	96; 194	9.61 (0.541)	8.55, 10.67
Age: ≥75 years	8; 20	5.50 (2.132)	1.32, 9.68
Site group: North	46; 92	9.22 (0.776)	7.70, 10.74
Site group: East	29; 62	10.94 (0.989)	9.00, 12.88
Site group: South	29; 60	7.76 (1.076)	5.65, 9.87
Baseline lumbar spine BMD T-Score: ≤–3	63; 143	9.89 (0.710)	8.49, 11.28
Baseline lumbar spine BMD T-Score: >–3 and ≤–2.5	32; 49	8.36 (0.713)	6.97, 9.76
Baseline lumbar spine BMD T-Score: >–2.5	9; 22	9.12 (2.472)	4.28, 13.97
Baseline vitamin D: ≤40ng/mL	98; 203	9.60 (0.533)	8.56, 10.64
Baseline vitamin D: >40ng/mL	6; 11	3.87 (2.201)	–0.44, 8.19
Baseline use of loading dose of vitamin D: Yes	92; 196	9.64 (0.551)	8.56, 10.72
Baseline use of loading dose of vitamin D: No	12; 18	6.61 (1.602)	3.47, 9.75

Data are shown for the full analysis set. Observed post-Baseline DXA BMD after treatment discontinuation not due to COVID-19 or alternative osteoporosis therapy was used in analysis as a treatment policy strategy and was imputed using the LOCF approach. Missing or out of window (exceeding 70 days since previous IMP) post-Baseline DXA BMD due to COVID-19 which led to treatment interruption was set to missing and imputed by multiple imputation under missing at random approach. BMD: bone mineral density; CI: confidence interval; COVID-19: coronavirus disease 2019; DXA: dual-energy x-ray absorptiometry; LOCF: last observation carried forward; LSM: least square mean; SE: standard error.

Supplementary Table 4. Serum trough concentrations of romosozumab (ng/mL) at final analysis of months 1 to 12 in participants initially randomized to romosozumab

Statistic	Baseline	Month 1	Month 3	Month 6	Month 7	Month 9	Month 12
n	216	203	164	142	137	137	135
Number of BLQs	215	0	1	0	1	0	1
Geometric mean	NC	3,147.63	3,792.80	4,367.43	4,742.83	4,453.18	4,454.64
95% CI	NC	2,878.72, 3,441.66	3,339.84, 4,307.20	3,877.70, 4,919.01	4,125.30, 5,452.79	3,942.58, 5,029.90	3,845.52, 5,160.24
Geometric CV (%)	NC	71.9	98.7	82.0	98.9	82.5	105.3
Mean	NC	3,817.13	4,866.17	5,510.95	6,073.18	5,575.89	5,764.88
SD	NC	2,401.80	3,253.54	3,818.48	4,126.75	3,821.50	3,683.51
Median	BLQ	3,273.00	4,137.50	4,428.50	5,118.00	4,544.00	5,211.00
Min	BLQ	384.0	BLQ	489.1	BLQ	211.3	BLQ
Max	261.00	15,650.0	18,590.0	24,290.0	29,590.0	24,090.0	19,560.0

Data are shown for the pharmacokinetic per-protocol set. BLQ: below limit of quantification; CI: confidence interval; CV: coefficient of variation; LLOQ: lower limit of quantification; max: maximum; min: minimum; NC: not calculable; SD: standard deviation.

Supplementary Table 5. Subject incidence of positively adjudicated cardiovascular events during double-blind placebo-controlled period

Positively adjudicated cardiovascular events<br>Preferred term (MedDRA v24.1)	Romosozumab<br>N=218<br>n (%) [#]	Placebo<br>N=109<br>n (%) [#]
**Any cardiovascular serious adverse event**	2 (0.9) [2]	0
Death	0	0
Cardiac ischemic event	2 (0.9) [2]	0
Acute myocardial infarction	1 (0.5) [1]	0
Coronary artery disease	1 (0.5) [1]	0
Non-coronary revascularisation events	0	0
Heart failure	0	0
Cerebrovascular event	0	0
Peripheral vascular events not requiring revascularisation	0	0

Data are shown for the safety set. n refers to the number of subjects reporting at least one TEAE in that category/with that preferred term. # refers to the number of individual occurrences of the TEAE. MedDRA: medical dictionary for regulatory activities; TEAE: treatment-emergent adverse event.

Supplementary Table 6. Subject incidence of positively adjudicated cardiovascular events during the overall period

MedDRA (v24.1)<br>Adverse event of interest<br>Preferred Term	Romosozumab/<br>Romosozumab<br>N=218<br>n (%) [#]	Placebo/<br>Romosozumab<br>N=109<br>n (%) [#]	Romosozumab<br>total<br>N=316<br>n (%) [#]
**Any TEAE for positively adjudicated cardiovascular**	3 (1.4) [3]	2 (2.0) [2]	5 (1.6) [5]
Acute myocardial infarction	1 (0.5) [1]	0	1 (0.3) [1]
Cerebral infarction	1 (0.5) [1][a]	1 (1.0) [1]	2 (0.6) [2]
Coronary artery disease	1 (0.5) [1]	0	1 (0.3) [1]
Lacunar infarction	0	1 (1.0) [1]	1 (0.3) [1]

Data are shown for the safety set. n refers to the number of subjects reporting at least one TEAE in that category/with that preferred term. # refers to the number of individual occurrences of the TEAE. [a]This event was serious, severe and fatal; the event was deemed not related to the investigational medicinal product by the investigator; the cardiovascular adjudication committee classified the cause of death as undetermined (i.e., not cardiovascular death nor death of a non-cardiovascular cause); therefore, this event was counted as positively adjudicated cardiovascular death. MedDRA: medical dictionary for regulatory activities; TEAE: treatment-emergent adverse event.
